# Synthesis and evaluation of a ^125^I-labeled iminodihydroquinoline-derived tracer for imaging of voltage-gated sodium channels^☆^

**DOI:** 10.1016/j.bmcl.2013.07.014

**Published:** 2013-09-15

**Authors:** Carlos Pérez-Medina, Niral Patel, Mathew Robson, Mark F. Lythgoe, Erik Årstad

**Affiliations:** a Department of Chemistry and Institute of Nuclear Medicine, UCL, 235 Euston Road (T-5), London NW1 2BU, United Kingdom; b Centre for Advanced Biomedical Imaging, UCL, 72 Huntley Street, London WC1E 6BT, United Kingdom; c UCL Cancer Institute, University College London, 72 Huntley Street, London WC1E 6BT, United Kingdom

**Keywords:** SPECT, Voltage-gated sodium channel, WIN17317-3, Iodine-125, Imaging

## Abstract

In vivo imaging of voltage-gated sodium channels (VGSCs) can potentially provide insights into the activation of neuronal pathways and aid the diagnosis of a number of neurological diseases. The iminodihydroquinoline WIN17317-3 is one of the most potent sodium channel blockers reported to date and binds with high affinity to VGSCs throughout the rat brain. We have synthesized a ^125^I-labeled analogue of WIN17317-3 and evaluated the potential of the tracer for imaging of VGSCs with SPECT. Automated patch clamp studies with CHO cells expressing the Na_v_1.2 isoform and displacement studies with [^3^H]BTX yielded comparable results for the non-radioactive iodinated iminodihydroquinoline and WIN17317-3. However, the ^125^I-labeled tracer was rapidly metabolized in vivo, and suffered from low brain uptake and high accumulation of radioactivity in the intestines. The results suggest that iminodihydroquinolines are poorly suited for tracer development.

## Graphical abstract

**Figure:**
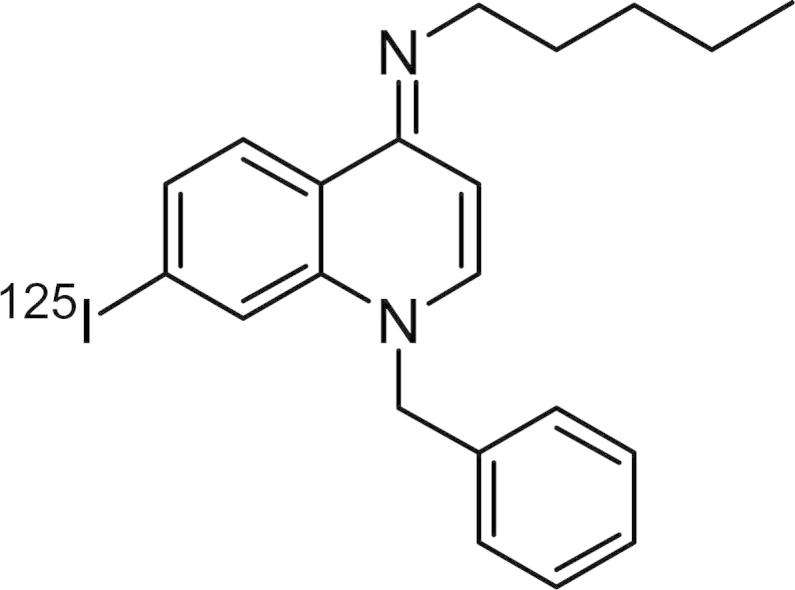

Voltage-gated sodium channels (VGSCs) are a family of transmembrane ion-channels responsible for the rising phase of action potentials in electrically excitable cells.^1^ Depolarization results in a conformational change of the channel from the resting non-conducting state to the open conducting state, which leads to selective influx of sodium ions. The ion channel then undergoes a further conformational change to the inactivated state, before it reverts back to its resting state.^2^ VGSCs can rapidly cycle through the resting, open and inactivated states, allowing neurons to fire the high-frequency trains of action potentials that are essential for fast neurotransmission. To date nine distinct alpha subunits of VGSCs (Na_v_1.1–Na_v_1.9) have been reported,^3^ each with their own distinct expression pattern in the central (CNS) and peripheral (PNS) nervous systems.^4^

VGSCs play a pivotal role in maintaining physiological conditions and higher cognitive functions, but also mediate hyperexcitation and neurodegeneration, and their dysfunction is implicated in a number of neurological diseases.^5–7^ Anomalous high-frequency repetitive spike firing, for instance, is known to take place during the spread of epileptic seizures,^8^ and there is an increasing body of evidence linking VGSC mutations with neuronal hyperexcitability in epilepsy.^9–11^ In fact, state-dependent VGSC blockers such as lamotrigine (**1**) and phenytoin (**2**) (Fig. 1), that can modulate the gating of sodium channels, are used as antiepileptic drugs (AEDs).^5^ Demyelinated sclerotic lesions overexpress VGSCs, and the resulting excessive influx of sodium ions is believed to cause axonal injury,^12^ which underpins the progression and symptoms of multiple sclerosis (MS).^13,14^ Interestingly, VGSC blockers have shown efficacy as neuroprotecting agents in animal models of MS,^15,16^ for treatment of migraine, as well as for psychiatric conditions such as schizophrenia, post-traumatic stress disorder and bipolar affective disorder.^5,17^

Development of radiotracers for noninvasive imaging of VGSCs with positron emission tomography (PET) and single photon emission computed tomography (SPECT) can potentially provide tools to study activation of neurological pathways, to investigate pathological processes, to diagnose disease, and to monitor the response to treatment.

WIN17317-3 (Fig. 1) was first reported to be a selective voltage-gated potassium channel blocker.^18,19^ However, in a subsequent study the binding of [^3^H]WIN17317-3 to rat brain synaptosomal membranes (*K*_d_ 2.2 ± 0.3 nM, *B*_max_ 5.4 ± 0.2 pmol/mg of protein) was shown to be insensitive to a number of potassium channel modulators and, on the contrary, could be blocked by several sodium channel ligands.^20^ Moreover, WIN17317-3 was found to inhibit sodium currents in CHO cells transfected with Na_v_1.2 (*K*_i_ 9 nM), and also blocked muscle sodium channels, and to a lesser degree sodium channels of the heart. Importantly, autoradiography of rat brain sections incubated with [^3^H]WIN17317-3 revealed high specific binding to sites that correspond with the known distribution of VGSCs in the CNS.^20^ The ability to depict VGSC distribution in vitro and the favorable physiochemical properties make WIN17317-3 appealing as a lead for tracer development. As part of our ongoing effort to develop tracers for imaging of VGSCs,^21^ we herein report the synthesis and biological evaluation of a ^125^I-labeled analogue of WIN17317-3.

The non-radioactive iodinated compound **10** (Scheme 1) was synthesized following the pathway described by Lin and Loo^22^ for the preparation of the key intermediate 4-chloro-7-iodoquinoline (**8**). The reaction of 3-iodoaniline (**3**) with diethyl ethoxymethylenemalonate (**4**) gave the acrylate **5**, which was converted to hydroxyquinoline **6** upon heating in diphenyl ether at 250 °C. Hydrolysis of the ester and subsequent decarboxylation afforded the intermediate **7** as a poorly soluble solid. Treatment of **7** with phosphorus oxychloride led to the formation of the chloroquinoline **8**, which was reacted with pentylamine to give the aminoquinoline **9**. Subsequent alkylation with benzyl bromide provided the non-radioactive reference compound **10** in 24% overall yield (five steps). Palladium(0)-catalyzed reaction of the iminodihydroquinoline **10** with hexamethylditin provided the corresponding labeling precursor **11** in 37% yield.

Iodo-destannylation of **11** with [^125^I]NaI in the presence of dilute hydrogen peroxide for 30 min at room temperature afforded the radioligand [^125^I]**10** in 58 ± 9% radiochemical yield (*n* = 7) with >99% radiochemical purity and a specific activity of 53.2 ± 7.1 GBq/μmol (*n* = 7). The identity of [^125^I]**10** was confirmed by co-elution with the non-radioactive reference compound **10**. The log *D*_7.4_ of [^125^I]**10** was measured to be 2.98 ± 0.08 (*n* = 3) using the traditional *n*-octanol shake flask method.^23^

The iodinated reference compound **10** was initially evaluated in an automated patch clamp assay using CHO cells transfected with the human Na_v_1.2 isoform (hNa_v_1.2, *SCN2A* gene).^24^ Comparison with WIN17317-3 (IC_50_ 2.2 ± 1.2 μM), which was included as a positive control, suggested that the blocking potency of the iodinated analogue **10** (IC_50_ 1.5 ± 0.5 μM) was fully retained. The discrepancy between our results and previously reported data for WIN17317-3 (IC_50_ 2.2 ± 1.2 μM vs 9 nM) is likely to reflect the proportion of non-protonated drug in the medium, as a physiological buffered bath solution was used in this study whereas the higher potency was recorded under basic conditions (pH 9.8).^20^ While the amino acid sequence of mammalian VGSCs have been largely preserved across the species, distinct pharmacological profiles have been reported for human and rodent isoforms.^4^ Hence, the potency of the iodinated iminodihydroquinoline **10** was further evaluated by displacement studies with [^3^H]BTX using rat brain homogenates (Ricerca Taiwan Ltd, Taiwan).^25^ The results obtained for WIN17317-3 (IC_50_ 8.1 nM; literature value of 25.6 nM^20^) and **10** (IC_50_ 21.1 nM) were comparable and largely in agreement with the patch clamp studies.

To evaluate the suitability of [^125^I]**10** for imaging VGSCs in vivo we measured the distribution of radioactivity after iv injection in female BALB/c mice (6–10 weeks old, 15–20 g) over a period of 1 h (5, 15, 30 and 60 min). The distribution of [^125^I]**10** in selected tissues is depicted in Figure 2. At the early time point a high uptake was observed in the liver and kidneys with later time points dominated by high uptake in the intestines. The initial brain uptake was low (0.48 ± 0.04% ID/g at 5 min) and did not show any sign of retention. The activity in the blood was also low and showed a moderate clearance, with 1.85 ± 0.17% ID/g at 5 min post-injection and 0.58 ± 0.04% ID/g at 60 min. Clearance from liver and kidneys was fast and was paralleled by an increased uptake in the intestines, consistent with hepatobiliary excretion.

In order to assess the metabolic stability of [^125^I]**10** the composition of radioactive species in plasma and brain tissue was analyzed by HPLC. In plasma, the fraction of the intact tracer [^125^I]**10** was 22.8% at 5 min, 12.5% at 15 min, and 5.2% at 30 min (*n* = 2), with the remaining activity observed as a highly polar metabolite (Fig. 3). Both the parent compound [^125^I]**10** and its radio-metabolite were also detected in the brain, however, low radioactivity levels made quantification of their relative concentrations difficult.

Finally, whole body SPECT/CT scans were recorded following injection of [^125^I]**10** in female BALB/c mice (4–10 weeks old). In agreement with the biodistribution studies, a high initial uptake of radioactivity was observed in the liver (0–15 min post-injection). At the later time points (15–35 min post-injection, Fig. 4), the images were dominated by high levels of activity in the intestines. Unfortunately, the uptake was too low to allow SPECT imaging of the regional distribution of [^125^I]**10** in the brain.

WIN17317-3 was attractive as a lead compound for tracer development as the tritiated derivative binds with low nanomolar affinity to VGSCs in rat brain tissue (*K*_d_ = 2.2 ± 0.3 nM) and allows imaging of VGSC expression on cryo-sections with in vitro autoradiography.^20^ Furthermore, substitution of the chlorine atom with radioiodine provided a versatile route to tracer candidates. Iodine-125 was chosen as labeling is straightforward and the long half-life (60 days) simplifies biological evaluation. The blocking efficiencies observed for the non-radioactive iodinated analogue **10** and WIN17317-3 in patch clamp studies, together with the data obtained from [^3^H]BTX displacement studies, suggest that iodine is well tolerated in the binding site and that **10** largely retained the biological properties of the parent compound. However, in vivo evaluation of [^125^I]**10** failed to demonstrate any binding that could be attributed to VGSC expression. Instead, rapid metabolism was observed accompanied by accumulation of radioactivity in the intestines. While iodinated tracers are prone to undergo enzymatic cleavage of the C–I bond to liberate iodide, the low uptake of radioactivity in the bladder, stomach and thyroid exclude de-iodination as a major metabolic route.^26^ The low metabolic stability of [^125^I]**10** is therefore likely to be related to the iminodihydroquinoline scaffold, and hence to also affect other derivatives of this class of compounds. It is plausible that the imino group undergoes hydrolysis in vivo; however, the retention time of the radio-metabolite observed is inconsistent with formation of the resulting ketone.

While the rapid metabolism is likely to have impaired the brain uptake, the low activity level in the brain at 5 min after injection of [^125^I]**10** points to other contributing factors, such as high protein binding or active extrusion by drug efflux pumps. It should also be noted that the parent compound is ionized at physiological pH, which may limit penetration through the blood–brain barrier.^27^ To rule out the possibility that the distribution pattern was dominated by specific binding to VGSCs expressed in the intestines,^28^ the composition of radioactivity in tissue samples was analyzed by radio-HPLC. However, the parent tracer [^125^I]**10** constituted only a minor component, with a number of other metabolites observed.

In conclusion, we have synthesised a ^125^I-labeled derivative of the VGSC blocker WIN17317-3. The non-radioactive reference compound was found to be a potent blocker of the human VGSC Na_v_1.2 isoform, and showed comparable potency to WIN17317-3 in [^3^H]BTX displacement studies. However, the ^125^I-labeled tracer failed to demonstrate specific VGSC binding in vivo, and suffered from poor metabolic stability and low brain uptake. Taken together, the results suggest that this class of compounds is poorly suited for tracer development.

## Figures and Tables

**Figure 1:**
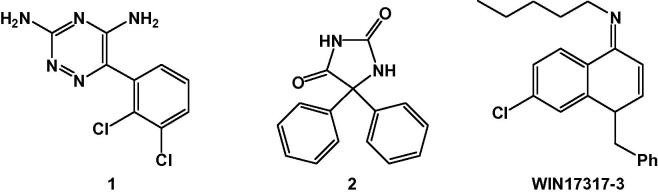
Structures of the state-dependent VGSC blockers lamotrigine (**1**), phenytoin (**2**) and WIN17317-3.

**Figure 2:**
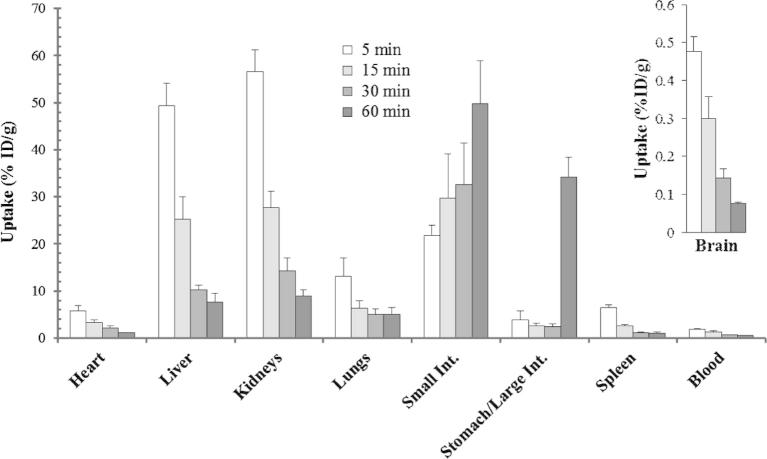
Tissue distribution of [^125^I]**10** expressed as % ID/g ± SD (*n* ⩾3).

**Figure 3:**
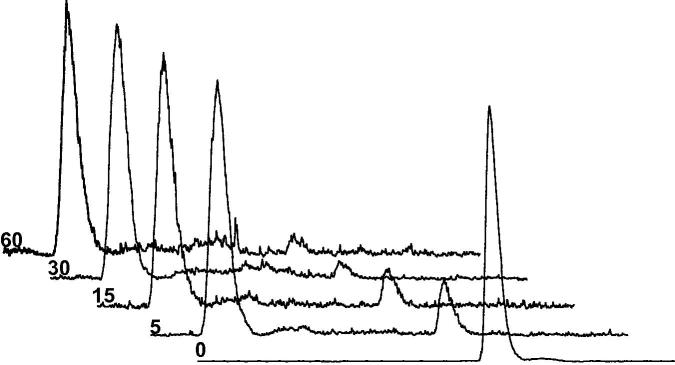
HPLC radioactivity profiles in plasma samples at 0, 5, 15, 30 and 60 min after injection of [^125^I]**10**.

**Figure 4:**
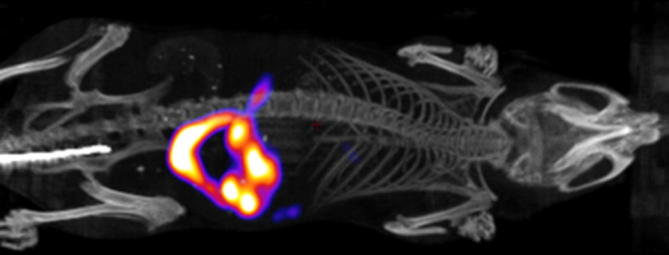
SPECT/CT summation image from 15 to 35 min after injection of [^125^I]**10**.

**Scheme 1:**
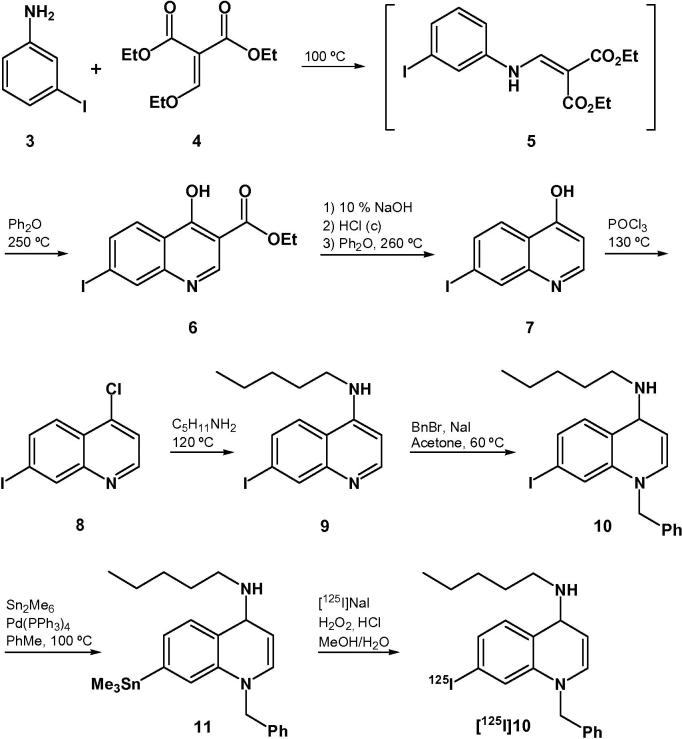
Synthesis of the iodinated radioligand [^125^I]**10**.

## Supplementary data

Supplementary data Materials and methods, synthetic procedures, selected ^1^H and ^13^C NMR spectra and selected radio-HPLC chromatograms.
