# Supplementary material for: Synthesis and evaluation of a 125I-labeled iminodihydroquinoline-derived tracer for imaging of voltage-gated sodium channels
Source: Bioorg Med Chem Lett. 2013 Sep 15;23(18):5170–3. doi: 10.1016/j.bmcl.2013.07.014 (PMC3764405; doi:10.1016/j.bmcl.2013.07.014)
Supplement: Supplementary data — Materials and methods, synthetic procedures, selected 1H and 13C NMR spectra and selected radio-HPLC chromatograms. [file mmc1.docx]

Supplementary Material

**Synthesis and Evaluation of a ^125^I-Labeled Iminodihydroquinoline-derived Tracer for Imaging of Voltage-Gated Sodium Channels**

Carlos Pérez-Medina^a^, Niral Patel^a,b^, Matthew Robson^b^, Mark Lythgoe^b^ and Erik Ǻrstad^a,*^

^a^ Department of Chemistry and Institute of Nuclear Medicine, UCL, 235 Euston Road (T-5), London NW1 2BU, United Kingdom.

^b^ Centre for Advanced Biomedical Imaging, UCL, 72 Huntley Street, London WC1E 6BT, United Kingdom.

^c^ UCL Cancer Institute, University College London, 72 Huntley Street, London WC1E 6BT, United Kingdom

Materials and Methods

**Chemistry.** All reagents were purchased from Sigma-Aldrich and were used without further purification. Column chromatography was performed on silica-gel (VWR BDH-Prolabo 40-63 µm). ^1^H and ^13^C NMR spectra were recorded at room temperature on Bruker Avance 300 or 500 instruments operating at the frequency of 300 or 500 MHz for ^1^H, and 75 or 125 MHz for ^13^C. All were internally referenced to the residual solvent peaks, CDCl_3_ (7.26 ppm), DMSO-*d_6_* (2.49 ppm) or CD_3_OD (3.31 ppm) for ^1^H, CDCl_3_ (δ 77.0 ppm), DMSO-*d_6_* (39.5 ppm) or CD_3_OD (49.1 ppm) for ^13^C. High resolution mass data were recorded on either a thermo Finnigan MAT900xp (CI/EI), Waters LCT Premier XE (ES) or a VG70-SE (FAB) mass spectrometers. HPLC analysis was performed with an Agilent 1200 HPLC system equipped with a 1200 Series Diode Array Detector and Fluorescence Detector.

**Radiochemistry.** [^125^I]NaI (3.7 GBq/mL, 643.8 MBq/mg) was purchased from Perkin Elmer Life and Analytical Sciences (331 Treble Cove Road, Billerica, MA 01862, USA) as a non-carrier-added solution in reductant free 10^-5^ M aqueous sodium hydroxide solution (pH 8-11).

**Radio-HPLC.** HPLC purification and analytical runs were carried out on a C18 Agilent Eclipse Plus column (4.6 x 150 mm, 5 μm) and monitored with a 254 UV detector and a Raytest Gabi NaI detector. The solvent systems used were water (0.1 % TFA, solvent A) and methanol (0.1 % TFA, solvent B) with a flow rate of 1 mL/min. Metabolite analysis was carried out on a C18 Agilent XDB column (4.6 x 150 mm, 5 μm) using water (0.1 % formic acid, solvent A) and methanol (0.1 % formic acid, solvent B) as solvent systems with a flow rate of 1 mL/min.

**LogD Determination.** LogD_7.4_ of [^125^I]**10** was determined using the *n*-octanol shake flask method.^1^ Briefly, a solution of radioligand in *n*-octanol (500 µL, presaturated with PBS 7.4) was mixed with an equal volume of PBS 7.4 (presaturated with *n*-octanol) and shaken for 15 minutes at 900 rpm, after which time the mixture was centrifuged at 10000 rpm for 5 minutes. The radioactivity present in both layers was measured in a gamma counter, and the *n*-octanol/PBS partition coefficient determined by dividing the radioactivity found in the *n*-octanol layer by that found in the PBS layer. The log value of this coefficient was then calculated. The process was repeated until a consistent value was obtained. The experiments were carried out in quadruplicate.

**Animals.** Female BALB/c mice were obtained from Charles River, UK. After arrival, the mice were allowed to acclimatise for at least 5 days in a room with constant temperature (21°C) and humidity (30%). All efforts were made to reduce the number of animals used, and all experiments were conducted in accordance with the UK Animals (Scientific Procedures) Act, 1986 and the European Community Council Directive, 1986.

**Metabolite Analysis.** Female BALB/c mice (6-10 weeks old and 15-20 g of weight) were injected with 2.4–3.4 MBq [^125^I]**10** in 120-300 µL saline solution (5 % ethanol) via their lateral tail vein. The mice were anesthetized with isoflurane (5% mixed with medical air at a flow rate of 2 mL/min). At 5, 15, 30 and 60 min post injection blood was drawn out by cardiac puncture and, immediately afterwards, animals were sacrificed by cervical dislocation and brains removed. Blood was collected into heparised tubes and centrifuged at 13000 rpm for 1 min to separate plasma; 300 μL of plasma were mixed with 1200 μL of cold acetonitrile, vortexed and centrifuged at 13000 rpm for 1 min. The supernatant was then transferred to a glass vial and mixed with 900 μL of water. Brain tissues were mixed with 1.5 mL acetonitrile/water 2:1 and homogenized, vortexed and centrifuged at 13000 rpm for 1 min. The resulting supernatant was then mixed with 500 μL of water and the resulting solution was centrifuged again as described above. Pellets and supernatants were separated and counted for radioactivity to determine recovery efficiency. An aliquot (1000 μL) of the supernatant obtained from the plasma and brain extracts was subjected to reversed-phase high-performance liquid chromatography (HPLC) analysis (Agilent C18 XDB column, 4.6 x 150 mm, 5 μm) using gradient elution from 50 to 90 % solvent B over 15 minutes at a flow rate of 1 mL/min. The recovery from plasma was measured to be 93.4 ± 1.0 % (n=3) and from brain 80.8 ± 0.1 % (n=3). Sample workup was identical as described above. Results are expressed as percentages of the total activity ± S.D.

**Biodistribution Studies.** Biodistribution experiments were conducted on female BALB/c mice (6-10 weeks old and 15-20 g of weight). The radiotracer (0.2-0.4 MBq) in 150-300 µL saline solution (5 % ethanol)) was administered via the lateral tail vein. The mice were anesthetized with isoflurane (5% mixed with medical air at a flow rate of 2 mL/min) at predetermined time points after administration (5, 10, 15, 30 and 60 min), blood was sampled through cardiac puncture, and the animals were then sacrificed by cervical dislocation. The radioactivity content in tissues of interest, (large and small intestine, stomach, kidneys, brain, bone (femur), liver, lungs, heart, skin, spleen, bladder, tail) was measured using a Wizard2 2470 Automatic Gamma Counter (Perkin Elmer). For each time point, 4 mice were used and the radioactivity uptake was calculated as the mean percentage injected dose per gram tissue ± S.D.

**SPECT Imaging.** Female BALB/c mice (4-10 weeks old) were injected with 20-35 MBq [^125^I]**10** in 200-300 µL saline solution (5-7 % ethanol) via the lateral tail vein and then anesthetized with isofluorane mixed with medical air (5% for induction, 2% for maintenance). Animals were immediately placed in prone position on a heated bed (37°C) under isofluorane anesthesia and scans were then performed using a NanoSPECT/CT small animal *in vivo* scanner (Bioscan Inc., 4590 MacArthur Blvd., NW, Washington D.C. 20007, USA). Whole body scans consisted of sixteen projections of varying duration and CT was recorded once the SPECT scans were finished. SPECT Images were reconstructed using HiSPECT software and In Vivo Scope software was used for CT image reconstruction.

Synthetic procedures

**4-Chloro-7-iodoquinoline (8).**^2^ A mixture of 3-iodoaniline (**3**, 5.75 g, 26 mmol) and diethyl ethoxymethylenemalonate (**4**, 5.67 g, 26 mmol) was stirred at room temperature until homogeneous, then heated at 100 °C for 3 hours under a continuous stream of argon to help remove ethanol. After this time, diphenyl ether (25 g) was added to the molten acrylate **5** and the mixture heated at 250-260 °C for 3 hours (a precipitate formed). After cooling down to room temperature, petroleum ether (50 mL) was added and the precipitate collected by filtration. The solid was washed with petroleum ether and allowed to dry to obtain **6**. This intermediate ester **6** (3.40 g, 10 mmol) was refluxed in 10 % aqueous sodium hydroxide for 1.5 hours and, after cooling down to room temperature, the solution was acidified with concentrated hydrochloric acid. The precipitate was filtered, washed with water and dried. A suspension of the resulting solid in diphenyl ether (10 g) was then heated at 250-270 °C for 6 hours until effervescence ceased. When cold, the mixture was diluted with petroleum ether and the solid filtered, washed with pentane and dried. Finally, a portion of this solid (crude 7-iodo-quinolin-4-ol, **7**, 0.50 g, 1.8 mmol) was reacted with phosphorus oxychloride (5 mL) at 130 °C for 2.5 hours. The excess POCl_3_ was removed under reduced pressure and the resulting oily residue treated with an ice-cold concentrated ammonia solution. After extracting with dichloromethane (2 x 20 mL), the organic layers were combined, dried over anhydrous magnesium sulphate and filtered. The brownish solid obtained after removing solvents was purified by column chromatography over silica gel eluting with petroleum ether/ethyl acetate 20:1, then 10:1 to furnish pure **8** as white crystals in 38 % overall yield. ^1^H-NMR (500 MHz, CDCl_3_) δ in ppm: 7.47 (d, ^3^*J*=4.7 Hz, 1H, H3), 7.87 (dd, ^3^*J*=8.9 Hz, ^4^*J*=1.4 Hz, 1H, H6), 7.91 (d, ^3^*J*=8.9 Hz, 1H, H5), 8.53 (d, ^4^*J*=1.4 Hz, 1H, H8), 8.75 (d, ^4^*J*=4.7 Hz, 1H, H2). ^13^C-NMR (125 MHz, CDCl_3_) δ in ppm: 97.0, 121.8 (CH), 125.4 (CH), 125.8, 136.5 (CH),138.8 (CH), 143.0, 149.7, 150.7 (CH). HRMS-FAB [M^+^]: m/z 288.9142 (calculated for C_9_H_5_NClI 288.9149).

**(7-Iodo-quinolin-4-yl)-pentyl-amine (9).** A solution of 4-chloro-7-iodoquinoline (**8**) in amylamine (2 mL) was heated at 120 °C for 24 hours, then allowed to cool to room temperature. The resulting solution was poured into ice/water (5 mL) and the precipitate collected by filtration, washed with water, then with pentane, and dried. The product was obtained as an off-white solid (0.34 g, 100 %).  ^1^H-NMR (500 MHz, CDCl_3_) δ in ppm: 0.93 (t, ^3^*J*=7.0 Hz, 3H, CH_3_), 1.41 (m, 4H, CH_2_CH_2_), 1.73 (m, 2H, CH_2_), 3.27 (t, ^3^*J*=7.4 Hz, 2H, CH_2_N), 5.05 (bs, 1H, NH), 6.40 (d, ^3^*J*=5.4 Hz, 1H, H3), 7.42 (d, ^3^*J*=8.8 Hz, 1H, H5), 7.63 (dd, ^3^*J*=8.8 Hz, ^4^*J*=1.7 Hz, 1H, H6), 8.36 (d, ^4^*J*=1.7 Hz, 1H, H8), 8.47 (d, ^4^*J*=5.4 Hz, 1H, H2). ^13^C-NMR (125 MHz, CDCl_3_) δ in ppm: 14.1 (CH_3_), 22.5 (CH_2_), 28.7 (CH_2_), 29.4 (CH_2_), 43.3 (CH_2_N), 95.0, 99.3 (CH), 117.9, 120.8 (CH), 133.1 (CH), 138.9 (CH), 149.5, 149.9, 151.8 (CH). HRMS-FAB [M^+^]: m/z 340.0423 (calculated for C_14_H_17_N_2_I 340.0431).

**(1-Benzyl-7-iodo-1*H*-quinolin-4-ylidene)-pentyl-amine (10).** A mixture of **9** (0.30 g, 0.88 mmol), sodium iodide (0.16 g, 1.2 eq) and benzyl bromide (0.12 mL, 1.1 eq) in acetone (4 mL) was heated at 60 °C for 5 hours. The resulting suspension was allowed to cool to room temperature and was then poured onto a mixture of 1 M sodium hydroxide (5 mL) and ice. After extracting with dichloromethane (2 x 15 mL), the organic layers were combined, dried over anhydrous magnesium sulfate and filtered. The residue obtained after evaporation of the solvents was chromatographed over silica gel eluting with ethyl acetate/petroleum ether first, then neat ethyl acetate and finally 2 % triethylamine in ethyl acetate to recover **10** as a yellow oil (0.24 g, 63 %). Purity was assessed by HPLC on an Agilent C18 Eclipse Plus column using water (0.1 % TFA; solvent A) and methanol (0.1 % TFA; solvent B) as eluents going from 10 % to 90 % B over 18 mins, at a flow rate of 1 mL/min. The retention time for **10** was 13.1 min and the purity > 98%. ^1^H-NMR (500 MHz, CDCl_3_) δ in ppm: 0.93 (t, ^3^*J*=7.1 Hz, 3H, CH_3_), 1.42 (m, 4H, CH_2_CH_2_), 1.72 (m, 2H, CH_2_), 3.31 (t, ^3^*J*=7.4 Hz, 2H, CH_2_N=), 4.98 (s, 2H, PhCH_2_N), 6.00 (d, ^3^*J*=8.1 Hz, 1H, H3), 6.90 (d, ^3^*J*=8.1 Hz, 1H, H2), 7.17 (d, ^3^*J*=7.2 Hz, 2H, Ph), 7.33 (m, 4H, H8, Ph), 7.43 (dd, ^3^*J*=8.5 Hz, ^4^*J*=1.5 Hz, 1H, H6), 8.15 (d, ^3^*J*=8.5 Hz, 1H, H5). ^13^C-NMR (125 MHz, CDCl_3_) δ in ppm: 14.3 (CH_3_), 22.8 (CH_2_), 30.2 (CH_2_), 31.0 (CH_2_), 50.3 (CH_2_N=), 55.3 (PhCH_2_N), 96.3, 99.6 (CH), 123.7 (CH), 125.6, 126.2 (CH), 127.2 (CH), 128.1 (CH), 129.2 (CH), 132.1 (CH), 135.7, 138.7 (CH), 139.9, 153.8. HRMS-CI [M+H^+^]: m/z 431.0979 (calculated for C_21_H_24_IN_2_ 431.0984).

**(1-Benzyl-7-trimethylstannanyl-1*H*-quinolin-4-ylidene)-pentyl-amine (11).** Compound **10** (85 mg, 0.2 mmol) was introduced in a two-neck round bottom flask equipped with a reflux condenser. The system was sealed and flushed with argon prior to the addition of degassed toluene (5 mL). Hexamethylditin (0.05 mL, 1.2 eq) and a solution of tetrakis(triphenylphosphine)palladium (0) (45 mg, 0.2 eq, in 2 mL of degassed toluene) were added then and the mixture stirred at 100 °C for 1.5 hours after which time it was allowed to cool to room temperature. The solution was decanted and the solid residue washed with dichloromethane. The toluene solution and the dichloromethane washings were combined, filtered and washed with 1 M NaOH (10 mL). The organic fraction was dried over anhydrous magnesium sulfate, filtered and concentrated under reduced pressure. The resulting residue was chromatographed on a silica gel column eluting with ethyl acetate/petroleum ether first, then neat ethyl acetate and finally 2 % triethylamine in ethyl acetate to get **11** as a yellow oil (40 mg, 37 %). ^1^H-NMR (500 MHz, CDCl_3_) δ in ppm: 0.17 (s, 9H, SnMe_3_), 0.93 (t, ^3^*J*=7.0 Hz, 3H, CH_3_), 1.42 (m, 4H, CH_2_CH_2_), 1.75 (m, 2H, CH_2_), 3.36 (t, ^3^*J*=7.4 Hz, 2H, CH_2_N=), 5.06 (s, 2H, CH_2_N), 6.03 (d, ^3^*J*=8.1 Hz, 1H, H3), 7.09 (d, ^3^*J*=8.1 Hz, 1H, H2), 7.11 (d, ^4^*J*=1.5 Hz, 1H, H8), 7.20 (d, 2H, Ph), 7.25-7.31 (m, 4H, H6, 3H Ph), 8.47 (d, ^3^*J*=8.7 Hz, 1H, H5). ^13^C-NMR (125 MHz, CDCl_3_) δ in ppm: -9.5 (SnMe_3_), 14.3 (CH_3_), 22.8 (CH_2_), 30.2 (CH_2_), 30.7 (CH_2_), 49.8 (CH_2_N=), 56.1 (CH_2_N), 98.7 (CH), 122.5 (CH), 124.4 (CH), 126.1, 126.3 (CH), 128.0 (CH), 129.1 (CH), 130.6 (CH), 136.4, 137.7, 139.4 (CH), 145.1, 154.8. HRMS-FAB [(M+H)^+^]: m/z 469.1652 (calculated for C_24_H_33_N_2_Sn 469.1666).

**Labelling of [^125^I]10.** Precursor **11** (50 µg) was placed in a glass vial and dissolved in methanol (100 µL). Hydrochloric acid (60 µL of a 0.4 M solution), hydrogen peroxide (50 µL of a 6 % v/v solution) and finally [^125^I]NaI (5-10 µL) were then added sequentially. The vial was sealed, shaken and left for 30 minutes at room temperature. The mixture was then diluted with MeOH/water 1:1 (500 µL) and injected into the HPLC system using a glass syringe. The compound was isolated using a C18 Agilent Eclipse Plus (4.6 x 150 mm, 5 µm) with the following eluent: water (0.1 % TFA) as solvent A and methanol (0.1 % TFA) as solvent B, going from 10 % B to 90 % B over 18 min. Retention time of [^125^I]**10** was 13.1 min. Integration of the UV absorption peak of [^125^I]**10** allowed the determination of the specific activity. The fraction containing the radioligand were diluted with water (10 mL) and passed through a Sep-Pak C18 light cartridge (Waters) using a 20 mL glass syringe. The loaded cartridge was washed with water (10 mL) and the radioligand was finally released with ethanol (0.5 mL, > 95 % recovery).

1. Arstad, E., Platzer S., Berthele A., Pilowsky L.S., Luthra S.K., Wester H.J., and Henriksen G. *Bioorg. Med. Chem.*, 2006, *14*, 6307.

2. Lin A.J. and Loo T.L. *J. Med. Chem.* 1978, *21*, 268.

Selected ^1^H and ^13^C NMR Spectra

**Figure 1.** ^1^H-NMR spectrum of compound **10**.

**Figure 2.** ^13^C-NMR spectrum of compound **10**.

**Figure 3.** ^1^H-NMR spectrum of compound **11**.

**Figure 4.** ^13^C-NMR spectrum of compound **11**.

Selected Radio-HPLC Chromatograms

**Figure 5.** HPLC chromatogram of the mixture of **10** and [^125^I]**10**, showing radioactivity (blue trace) and UV absorption at 254 nm (red trace), and demonstrating co-elution (2 % offset between signals).

min

0

2

4

6

8

10

12

14

16

**mAU**

0

20

40

60

80

100

**10**

[^125^I]**10**

**Figure 6.** HPLC radioactivity profile of an intestinal sample at 90min after injection of [^125^I]**10**.

0

**mAU**

min

18

1000

1200

1400

2

4

6

8

10

12

14

16

0

200

400

600

800

[^125^I]**10**
